# X-ray Characterization of Conformational Changes of Human Apo- and Holo-Transferrin

**DOI:** 10.3390/ijms222413392

**Published:** 2021-12-13

**Authors:** Camila Campos-Escamilla, Dritan Siliqi, Luis A. Gonzalez-Ramirez, Carmen Lopez-Sanchez, Jose Antonio Gavira, Abel Moreno

**Affiliations:** 1 Instituto de Química, Universidad Nacional Autónoma de Mexico, Av. Universidad 3000, Ciudad Universitaria, Ciudad de Mexico 04510, Mexico; camila.cescamilla@gmail.com; 2 Istitituto di Cristallografia (IC), National Research Council (CNR), Via Amendola 122/O, 70126 Bari, Italy; 3 Laboratorio de Estudios Cristalográficos, Instituto Andaluz de Ciencias de la Tierra, C.S.I.C. University of Granada, Avenida de las Palmeras No. 4, 18100 Armilla, Granada, Spain; luis.gonzalez@csic.es (L.A.G.-R.); carmen@iact.ugr-csic.es (C.L.-S.); jgavira@iact.ugr-csic.es (J.A.G.)

**Keywords:** Small-Angle X-ray Scattering, X-ray Crystallography, human serum transferrin, conformation change, pH-dependence

## Abstract

Human serum transferrin (Tf) is a bilobed glycoprotein whose function is to transport iron through receptor-mediated endocytosis. The mechanism for iron release is pH-dependent and involves conformational changes in the protein, thus making it an attractive system for possible biomedical applications. In this contribution, two powerful X-ray techniques, namely Macromolecular X-ray Crystallography (MX) and Small Angle X-ray Scattering (SAXS), were used to study the conformational changes of iron-free (apo) and iron-loaded (holo) transferrin in crystal and solution states, respectively, at three different pH values of physiological relevance. A crystallographic model of glycosylated apo-Tf was obtained at 3.0 Å resolution, which did not resolve further despite many efforts to improve crystal quality. In the solution, apo-Tf remained mostly globular in all the pH conditions tested; however, the co-existence of closed, partially open, and open conformations was observed for holo-Tf, which showed a more elongated and flexible shape overall.

## 1. Introduction

Human serum transferrin (Tf), also known as serotransferrin, is a ~80 kDa glycoprotein that transits blood plasma, whose function is to maintain iron homeostasis by transporting iron from its absorption site to every cell through the blood vessels. Its structure consists of two homologous lobes, an N-terminal lobe and a C-terminal lobe, each subdivided into two domains, in between which a metal-binding site is found, thus allowing Tf to bind two ferric ions per molecule [1,2]. The transferrin completes a cycle in which it transits the bloodstream as holo-Tf (iron-loaded form) and binds to the transferrin receptor (TfR) expressed on endothelial cells. The Tf–TfR complex formed is internalized in endosomes, where the pH is more acidic (5.5) than in the bloodstream (7.4); it is suggested that this pH decrease promotes a conformational change that induces iron release from the metal-binding site of transferrin. Following iron release, apo-Tf (iron-free form) is detached from the TfR and recycled into the bloodstream, where it captures more ferric iron [3,4].

Transferrin’s mechanism of iron transport draws special attention because it occurs in endothelial cells of the blood–brain barrier. These tightly bound cells restrict the influx of material from the bloodstream, limiting the possibilities of treatment for various neurological diseases; however, transferrin is able to cross this barrier thanks to receptor-mediated endocytosis [5,6]. Moreover, transferrin is not limited to binding iron, and it may bind other molecules of therapeutic interest [7,8,9,10,11]. Therefore, it is an attractive candidate for targeted drug delivery. To determine the feasibility of such a system, a thorough structural and bioanalytical characterization of transferrin is essential. X-ray crystallography is one of the most widely used approaches for structure determination, and several Tf models have been obtained so far [1,2,12,13,14]; however, further research is necessary to generate models that provide deeper knowledge for potential biomedical applications.

Although the behavior of a macromolecule in its physiological environment is not the same as it is in vitro, it is helpful to know about it to fine-tune experimental setups. The aim of this contribution is to provide insight into the structural complexity of native human serum transferrin and the challenges that go along with it. When crystallizing transferrin, two important factors must be considered: its great conformational flexibility and the fact that its mechanism of iron release is pH-dependent [2,3,6,15] and has an important impact on its conformational states [16]. In this work, we first summarize the efforts to improve crystal quality which yielded little success in solving the structure via crystallographic methods at a very high resolution. Considering that we are dealing with a highly flexible system, we employed one of the most powerful techniques for obtaining structural information of molecules in a solution: Small-Angle X-ray Scattering (SAXS). Therefore, we further characterized the system using SAXS, exploring not only protein concentration, but also the behavior of the apo- and holo-Tf at three physiologically and experimentally relevant pH conditions. From this study, we can propose conformational details of the mechanism of pH-dependent iron release from transferrin. 

## 2. Results and Discussion

Numerous crystals of apo- and holo-Tf have been grown by the classic vapor-diffusion technique, showing great optical properties and well-defined morphology (Figure S1), but limited diffraction resolution after many trials. This might be due to several factors that affect crystal packing, as well as a high sensitivity to temperature and pH alterations. To optimize the conditions for apo-Tf and holo-Tf crystal growth and X-ray diffraction for structural determination, some dedicated effort has been made, both chemically and physically. We explored the crystallization space chemically by including sets of new additives. Crystals obtained by vapor diffusion were protected with a variety of cryo-protection strategies, from naked crystal diffraction to several standard cryo-protectants tested at different concentrations and soaking times. To provide better control of nucleation and crystal growth, we turned to several non-conventional crystallization techniques [17,18,19,20], namely counter-diffusion in agarose gels in several of its possible setups, which also allows the exploration of the supersaturation rate. These efforts have yielded good-looking crystals, but their stability is compromised once they are removed from their growth medium, making it impossible to proceed to X-ray diffraction. In view of these results, the time it would take to obtain crystals with an appropriate size and stability for X-ray diffraction through these techniques’s results is impractical.

After exploring a plethora of strategies, a crystallographic model of apo-Tf has been constructed from X-ray diffraction data from one of the best crystals grown under the conventional vapor-diffusion technique. The data collection and refinement statistics for our apo-Tf model, deposited under the 7Q1L PDB code, are summarized in Table 1.

The data for our apo-Tf model are good enough for visualizing the general protein structure, allowing the location of two NAG moieties bound to residue Asn413. Some extra density could be observed after the second NAG element, which points to a potential elongation of the glycosyl chain that could be the key factor hampering crystal quality improvement. As has been reviewed, glycosylation does not necessarily prevent crystallization—it may even help—but the resolution limit and diffraction quality may suffer [21]. Superposition with chain A of glycosylated and non-glycosylated apo forms of human transferrin (PDB ID 2HAV and 2HAU) give RMSD values of 0.56 Å and 0.70 Å, respectively. However, the resolution is still limited in its ability to provide insight into the functional details. Therefore, this called for alternative structural determination strategies that do not require a crystal sample. One of these techniques is Small-Angle X-ray Scattering (SAXS), a low-resolution technique that provides structural information of proteins in quasi-native conditions, as they are studied in a solution [22]. The experimental and calculated SAXS details for both apo- and holo-Tf forms are summarized in Table 2.

**Figure 1:**
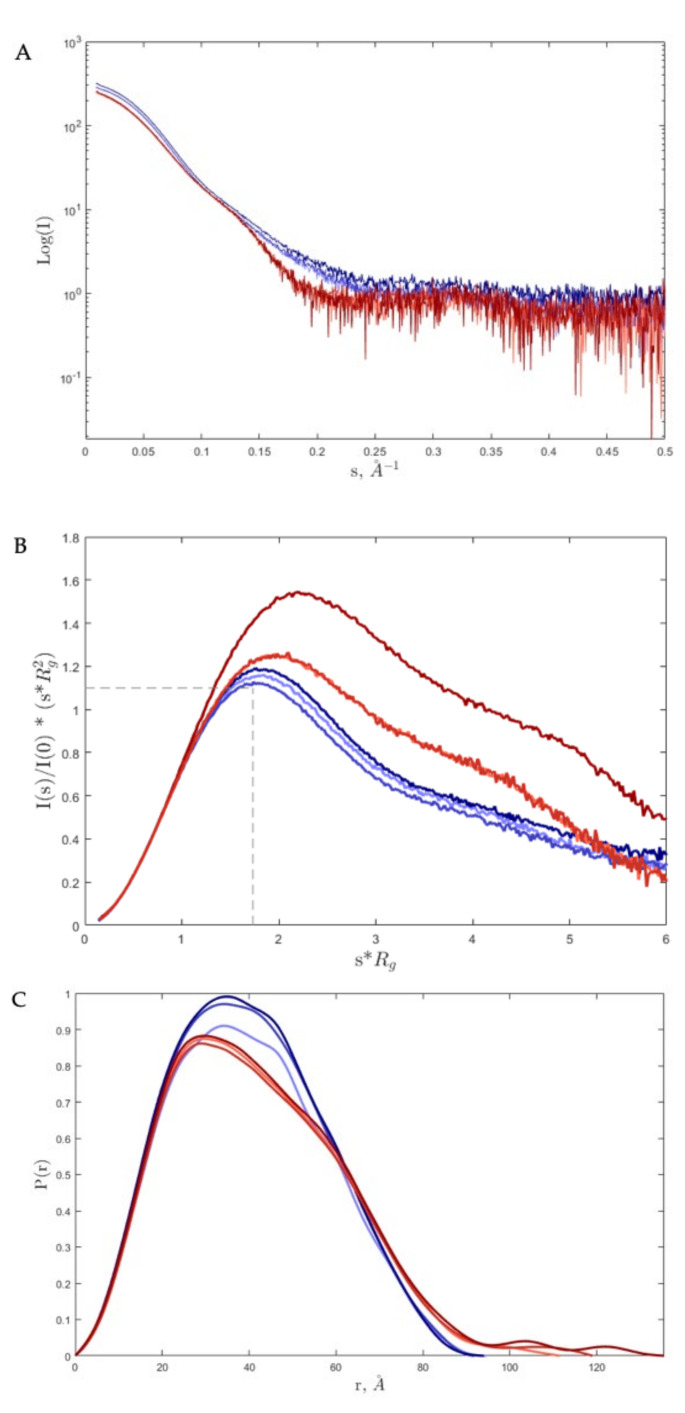
(**A**). Log10 SAXS intensity versus scattering vector, s. The plotted range represents only positive data within the specified s-range. (**B**). Dimensionless Kratky plot. The maximum value (intersection of dashed black lines) at Guinier–Kratky point (1.732, 1.1) corresponds to the main peak position for globular particles. (**C**). Pair-Distance, P(r), Distribution Function. The maximum dimension, D_max_, is the largest non-negative value that supports a smooth distribution function. The curves are overlapped; the red and blue curves correspond to iron-loaded transferrin (holo-Tf) and iron-free transferrin (apo-Tf), respectively. The color gradient for both from light to dark represents the change of pH from 8.0 to 5.5, correspondingly.

SAXS analysis of the one-dimensional SAXS experimental curves (Figure 1A) was initially performed to judge the quality of the data and to obtain basic structural information related to the size and shape of the studied proteins. One such structural parameter is the radius of gyration (R_g_) calculated from the slope of the Guinier plot described as ln(Is) vs. s^2^, where s = 4 πsin(θ)/λ is the scattering vector (2θ is the scattering angle and λ is the wavelength) [23]. For globular proteins, this plot is expected to be linear at low s, corresponding to values of s*R_g_ into Guiner zone (1.0-1.3). The linearity of the Guinier plot is considered a quality measurement of the data, but it does not ensure the ideality of the sample. Guinier plots for apo-Tf and holo-Tf were linear (Figure S2), and the R_g_ value had an average value of 31 Å and 33 Å (see Table 2 for more details) for apo and holo, respectively, and no differences were noted at different pH values.

The dimensionless Kratky plot was used to investigate the flexibility (and shape) of the proteins (Figure 1B). The maximum value of 1.104 at √3 (dashed black line) corresponds to a globular and compact protein, such as the bovine serum albumin (BSA) protein used as a standard in these experiments. For holo-Tf at different pH values (red curves), the maxima were shifted to the right (larger than √3) and were higher than the standard, which denoted a well-folded but asymmetric shape. For apo-Tf (blue curves), no differences were observed at different pH values, compared to holo-Tf, especially at the lowest level of pH. Furthermore, in Table 2, the values for the Porod coefficient are shown, and it can be noted that for the holo form, the protein is more flexible than for apo. In conclusion, by using R_g_, D_max_, Kratky plot, and Porod coefficient, it is possible to see that holo-Tf is more elongated and more flexible, and this is more evident at low pH.

The maximum size of a protein (Dmax) can be obtained from analysis of the SAXS data by means of the Pair-Distance Distribution Function (P(r)) (Figure 1C), which corresponds to the distribution of distances between all the electrons within the protein. The Pair-Distance Distribution Function is obtained using the Indirect Fourier Transformation [24], with a trial-and-error procedure at the end of which the obtained D_max_ corresponds to the smoothest and most positive distribution. Differences in the D_max_ of a protein relate to conformational changes. Additionally, it is possible to calculate the R_g_ from the Pair-Distance Distribution Function and compare its value with that estimated from the Guinier plot. In the case of holo-Tf, differences in D_max_ were observed as the pH value lowered, suggesting a conformational change induced by an acidic environment (Table 2). For apo-Tf, no differences were observed at different pH values with respect to holo-Tf. For the latter, P(r) distribution (Figure 1C), as well as R_g_ and D_max_ values (Table 3), showed a more elongated molecule, particularly at the lowest pH.

**Figure 2:**
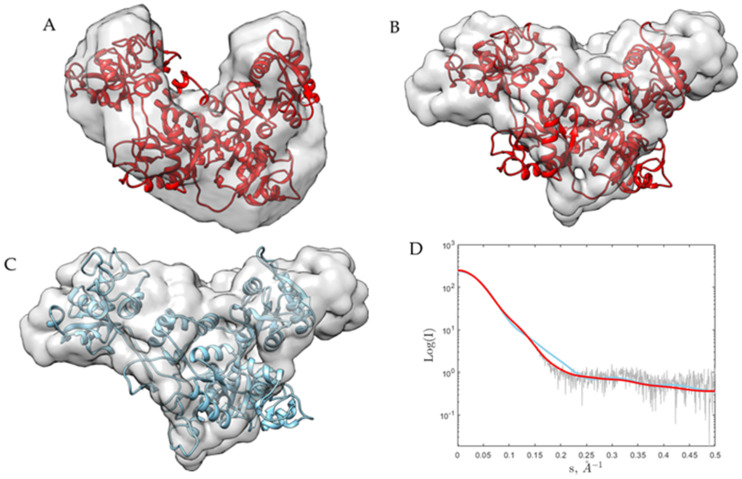
(**A**) and (**B**), 2HAV (chain A) model fit into DAMMIN (ab initio SAXS modeling) for apo-Tf at pH 8.0 and holo-Tf at pH 5.5, respectively. (**C**), 2HAV (chain A) model refined by SREFLEX fit into DAMMIN (ab initio SAXS modeling) for holo-Tf at pH 5.5. (**D**), Fitting of the calculated SAXS for the 2HAV model (chain A) (red) and after the refinement (SREFLEX) (cyan) scattering curves for holo-Tf at pH 5.5 compared to the experimental scattering signal (blue).

For all the data sets, SAXS modeling was performed by using an ab initio reconstruction of a protein structure by simulated annealing using a single-phase dummy atom [25]. For apo-Tf, independently of the pH, the reconstructions are the same (superposed), and the 2HAV (chain A) model fit very well into it. For simplification, in Figure 2A, this is shown only for pH 8.0. On the contrary, for holo-Tf, the models showed differences (more elongated) with respect to the apo-Tf models (more globular) and especially, as expected from global parameters (R_g_ and D_max_), for pH 5.5. For simplification, the last case (holo-Tf at pH 5.5) is shown in Figure 2B, indicating that the holo-Tf changed its conformation with respect to the apo-Tf and that this depends on pH changes. We used the SREFLEX program [26], which uses normal mode analysis (NMA) to estimate the flexibility of high-resolution models of biological macromolecules and improves their agreement with experimental SAXS data. In Figure 2C, we show the fitting into the DAMMIN model of the refined and improved (the χ^2^ is decreased from 33.5 to 1.74) holo-Tf model by SREFLEX.

**Figure 3:**
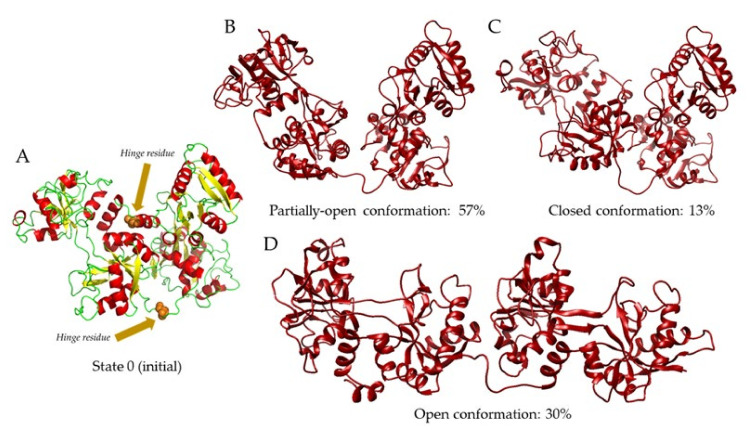
SAXS-based pseudo-atomic modeling using MultiFoXS for holo-Tf at pH 5.5. Multi-state models at different pH values were obtained after sampling over 10,000 conformations using the 2HAV (chain A) structure as a starting model and Thr371 and Leu671 as hinge residues (shown by spheres), corresponding to State 0 (**A**). For State 3, three conformations were observed in the following proportions: partially open, 57% (**B**); closed, 13% (**C**); open, 30% (**D**).

To further investigate the pH dependence of transferrin, we employed a SAXS-based pseudo-atomic modeling approach using the MultiFoXS server [27]. This technique can be used for the structural characterization of flexible proteins in a solution if a high-resolution structure or a comparative model of the studied protein is available. The procedure consists of a low-resolution rigid-body fitting to the experimental data that includes flexibility between the folded domains that make up the structure. This is performed by sampling random conformations along flexible residues and considering that an ensemble of multiple conformations contributes to a single observed SAXS profile. As a starting model, we used chain A from the 2HAV structure (Figure 3A, State 0) after removing the ligand, and hinge residues (T336 and L671) were identified by the HingeProt program [28]. The MultiFoXS server sampled over 10,000 conformations, using the hinge residues as above, calculated their SAXS profiles, and scored multi-state models according to their fitting to the experimental profile. The state number corresponds to the number of possible conformations. For instance, State 0 corresponds to the starting model; then, from State 1 to State 2, the fitting of the weighted calculated profile for two conformations is lowered to the χ^2^ value (better fitting). The same is true from State 2 to State 3. Therefore, the higher the number of states, the higher the flexibility of the model. In Table 3, all the data are summarized. For each conformation, the value of the fraction, Rg, and Dmax are indicated for each State, as well as the corresponding χ^2^ fitting value. In Figure 3B–D, the conformations for State 3 (three conformations) of holo-Tf at pH 5.5 are shown, with their contribution shown as a percentage (instead of the fraction as in Table 3), indicating the two hinge residues (Figure 3A) in the initial model (State 0). We did not observe any conformational changes for the apo form. Indeed, 90% (Table 3) of the available conformations are populated by the “closed” conformation, corresponding to the initial model.

The SAXS analyses performed here at different pH conditions confirm that there are different conformations co-existing in the solution. Although for apo-Tf there are no major differences observed, holo-Tf shows a predominant population of a partially open conformation, besides the open and closed conformations, as evidenced by the R_g_ and D_max_ values for the three-state model at all pH values (Figure 3). These results also show that holo-Tf is more elongated and flexible than the apo-form, suggesting that the presence of iron has an impact on the stability of transferrin in the solution. Furthermore, the partially open conformation in holo-Tf (Table 3) may suggest a monoferric state, wherein transferrin has only one of its lobes loaded with iron.

An interesting observation when analyzing the holo-Tf crystals grown for crystallographic analysis is that they lose the coloration that is indicative of the presence of iron, as observed when holo-Tf is in a solution. This has led us to hypothesize that these crystals correspond to a polymorph of apo-Tf, and iron is probably lost during the crystallization process, as the optical properties of transferrin differ depending on the amount of iron present within the protein [29]. This notion is also supported by the differences between apo-Tf and holo-Tf observed with SAXS.

## 3. Materials and Methods

### 3.1. Purification of Transferrin

Human serum transferrin was commercially obtained (Sigma-Aldrich, St. Louis, MO, USA) in both its iron-free (apo) and iron-loaded (holo) forms. The same conditions were used for purifying both. The proteins were suspended in a buffer containing 50 mM TRIS-HCl pH 8.0 with 20 mM NaHCO_3_ and filtered through a 0.22 μm pore. The purification was accomplished by ion-exchange chromatography in a HiTrap Q HP 5 mL column using a linear gradient from 0 to 100% high salt buffer (50 mM TRIS-HCl pH 8.0 with 20 mM NaHCO_3_ and 1.0 M NaCl). After elution, apo- and holo-Tf were dialyzed against 15 mM HEPES pH 8.0 with 20 mM NaHCO_3_ and 50 mM NaCl using a 6–8 kDa MWCO membrane, at 4 °C for 2 h, then other 2 h with a fresh buffer, and finally overnight with a fresh buffer. Finally, apo and holo were concentrated by centrifugation in Amicon tubes at 3000× *g* in 15-min cycles at 4 °C.

### 3.2. Crystallization and Macromolecular Crystallography

Transferrin crystals were grown at a stock concentration of 20 mg mL^−1^ using Index screen #88 (Hampton Research, Aliso Viejo, CA, USA) condition as the precipitant solution, which consists of 0.2 M ammonium citrate tribasic, pH 7, and 20% PEG 3350. Crystals were grown at 18 °C by the vapor-diffusion method as previously standardized [13,30]. 

Prior to data collection, crystals were subject to cryo-preservation by being flash cooled in liquid nitrogen and then stored. Because crystal quality was the main issue, several protocols were assayed, including the use of several cryoprotectants (i.e., glycerol, PEG 200, MPD, etc.) at different concentrations and soaking times, the use of naked crystals, or long soaking (24 h) for crystals obtained in capillaries by the counter-diffusion technique. The crystal quality of a total of 103 different samples was evaluated from X-ray diffraction data collected from beamlines XALOC of ALBA (Barcelona, Spain), ID-30B, ID23-1, ID30A-3 of ESRF (Grenoble, France), as well as from SSRL [31,32] synchrotron sources.

### 3.3. X-ray Diffraction

The X-ray data collection was performed at the microfocus beamline BL-14-1 at the Stanford Synchrotron Radiation Lightsource, SLAC National Accelerator Laboratory (Menlo Park, CA, USA). The crystals were cryo-protected with 30% (*v*/*v*) glycerol and immediately put into the X-ray beam under cryogenic conditions at 100 K. The wavelength of incident X-rays was 1.13 Å with an Eiger 16M detector. Data-collection strategies included high-redundancy data, and each sample was rotated in 0.25° increments. The HKL3000 [33] suite and XDS [34] were used to process initial data that were further merged, scaled, and reduced with the Aimless [35] of the CCP4 software suite [36]. Molecular replacement was achieved using chain A of the apo-Tf model PDB ID 2HAV as the search model in Molrep [37]. Initial refinement began with Refmac [38] and was finalized with phenix.refine [39]. Final model quality was assessed with MolProbity [40], and the files were prepared for deposition with PDB extract [41]. Refinement statistics and quality indicators are summarized in Table 1.

### 3.4. Small-Angle X-ray Scattering (SAXS)

SAXS experiments for transferrin were carried out at BM29 [42,43] beamline at the European Synchrotron Radiation Facility (ESRF, Grenoble, France). The wavelength of incident X-rays was 1 Å, and the Pilatus3 2M detector, in vacuum, was placed 2.867 m from the sample, leading to a scattering vector range from 0.025 to 6 nm^−1^. To avoid radiation damage, samples with a volume of 40 µL were measured using a robotic sample handler [44] in flow-through mode, collecting over 10 frames lasting 1 s for each sample. Frames were automatically checked for radiation damage, and those not displaying any radiation damage were then averaged [45]. Before and after each sample, buffer scattering was collected and subtracted from sample scattering. The buffer consisted of 15 mM HEPES, 20 mM NaHCO_3_, and 50 mM NaCl, tested at pH 8.0, 7.0, and 5.5. To assess concentration effects, a dilution series consisting of 2 concentrations (2.5 and 5 mg mL^−1^), for both Tf-apo and Tf-holo samples, was measured. The stock protein sample was at a concentration of 20 mg mL^−1^ and it was diluted up to 5 mg mL^−1^ and 2.5 mg mL^−1^, using the buffer corrected at pH 7.0 and 5.5 for the tests on these pH. We did not check the final pH of the solution, but we calculate that during the dilution, the pH should not be significantly different from the expected value. Since the scattering curves for Tf did not display any concentration dependence at the highest concentrations (Figures S3–S8), this concentration (5 mg mL^−1^) was used in our analysis. Merging, subtracting, and subsequent analysis were performed using PRIMUS [46] and Scatter [47] software. The SAXS modeling was performed by using DAMMIN [25], SREFLEX [26], and MultiFoXS [27] software. Theoretical scattering curves were calculated and compared to experimental SAXS profiles using CRYSOL [48]. Modeling and fitting figures were obtained with Chimera [49].

## 4. Conclusions

The Small-Angle X-ray Scattering data clearly showed a strong pH dependency and open-structured conformation at different times and pH values. Static snapshots that we attempted to derive from X-ray diffraction data were hampered by intrinsically low crystal quality and/or high sensitivity to crystal manipulation. We can conclude that the glycosylation is perhaps the main reason that all the efforts to improve crystal quality were not successful, along with the high flexibility of the structure, as shown in the SAXS experiments in the solution. From this study, we can propose conformational details of the mechanism of pH-dependent iron release from transferrin, including a monoferric intermediate state, which will have important biomedical research impacts on the transport of iron or ligands against brain diseases.

## Figures and Tables

**Table 1:** Data collection and refinement statistics (values in parentheses are for highest-resolution shell).

Protein	Apo-Transferrin
Ligand	NAG
PDB identifier	7Q1L
Data collection	
Beamline	BL14-1
Space Group	P 2_1_ 2_1_ 2_1_
Cell dimensions	
a, b, c (Å)	87.63, 102.15, 199.97
ASU	2
Resolution (Å)	37.24– 3.0 (3.107– 3.0)
R*_merge_* (%)	9.32 (116.0)
I/σ_I_	12.13 (1.16)
Completeness (%)	99.81 (99.82)
Unique reflections	36660 (3606)
Multiplicity	7.3 (8.0)
CC_(1/2)_	0.999 (0.559)
Refinement	
Resolution (Å)	37.24– 3.00
R*_work_*/R*_free_* (%)	22.03 / 25.01
No. atoms	10716
Protein	10592
Ligands	235
Water	7
B-factor (Å^2^)	120.52
R.m.s deviations	
Bond lengths (Å)	0.005
Bond angles (^0^)	0.85
Ramachandran (%)	
Favored	92.95
Allowed	6.75
Outliers	0.30

**Table 2:** Experimental and calculated SAXS data.

Data Collection Parameters						
Beamline	BM29, ESRF					
Detector	Pilatus3 2M in vacuum					
Beam size (mm)	0.2 × 0.2					
Energy (keV)	12.5					
	
Sample-to-detector distance (mm)	2.867					
s range (A^−1^)	0.0025–0.6					
Exposure time (s)	1					
Temperature (K)	293					
Data collection mode	In batch, automated sample changer					
	**apo-Tf**			**holo-Tf**		
**Structural parameters**	pH 8.0	pH 7.0	pH 5.5	pH 8.0	pH 7.0	pH 5.5
Concentration range (mg mL^−1^)	5	5	5	5	5	5
*s* Interval for Fourier inversion (Å^−1^)	0.014–0.258	0.015–0.258	0.017–0.258	0.018–0.247	0.021–0.249	0.015–0.248
R_g_ (from P(r)) (Å)	31.2 ± 0.02	31.2 ± 0.02	31.2 ± 0.02	33.0 ± 0.05	33.2 ± 0.05	34.2 ± 0.06
R_g_ (from Guinier approximation) (Å)	31.1 ± 0.00	31.1 ± 0.00	31.1 ± 0.01	32.4 ± 0.01	33.4 ± 0.01	33.7 ± 0.01
*s*R_g_ limits (from Guinier approximation)	0.44–1.29	0.44–1.28	0.21–1.30	0.56–1.28	0.66–1.30	0.48–1.30
D_max_ (Å)	94.2	96.4	98.4	131.0	135	146
Porod coefficient	3.7	3.7	3.7	3.5	3.0	3.0
Porod volume estimate (nm^3^)	104	106	106	106	103	105
Model excluded volume (nm^3^)	119					
Molecular Mass (kDa) from:						
Porod volume	66.0					
Excluded volume (×0.5)	60.0					
From sequence	77.0					
Modeling Ambiguity	2.1 (might be ambiguous)			2.4 (might be ambiguous)		
**SASBDB code**	SASDMP7	SASDMN7	SASDMM7	SASDMS7	SASDMR7	SASDMM7
**SAXS software employed**						
Primary data reduction			ESRF online software tools			
Data processing			ScÅtter IV/ATSAS 3.0.4			
Computation of model intensities			CRYSOL (ATSAS)			
Modeling			DAMMIN (ATSAS), SREFLEX (ATSAS), MultiFoxs			

**Table 3:** Experimental and calculated data for apo- and holo-Tf at three different pH values.

apo-Tf					
pH 8.0					
State	Conformation	Fraction	R_g_	D_max_	χ^2^
0	0	1	29.5	102.4	6.5
1	1	1	30.4	103.4	3.6
2	1	0.9	29.9	103.2	1.6
	2	0.1	36.7	127.1	
					
pH 7.0					
State	Conformation	Fraction	R_g_	D_max_	χ^2^
0	0	1	29.5	102.4	5.8
1	1	1	30.2	103.2	3.2
2	1	0.88	29.9	103.4	1.2
	2	0.12	33.8	119.6	
					
pH 5.5					
State	Conformation	Fraction	R_g_	D_max_	χ^2^
0	0	1	29.5	102.4	8.5
1	1	1	30.1	103.1	6.3
2	1	0.91	30.1	103.1	1.4
	2	0.09	33.8	119.9	

**holo-Tf**					
pH 8.0					
State	Conformation	Fraction	R_g_	D_max_	χ^2^
0	0	1	29.5	102.4	28.6
1	1	1	31.7	106.4	7.3
3	1	0.5	31.6	103.0	4.1
	2	0.26	29.8	102.5	
	3	0.24	37.31	127.7	
pH 7.0					
State	Conformation	Fraction	R_g_	D_max_	χ^2^
0	0	1	29.5	102.4	45.6
1	1	1	31.9	109.6	13.2
3	1	0.44	31.9	104.0	6.43
	2	0.35	30.4	103.9	
	3	0.21	37.5	132.9	
					
pH 5.5					
State	Conformation	Fraction	R_g_	D_max_	χ^2^
0	0	1	29.5	102.4	33.6
1	1	1	31.9	109.9	4.59
3	1	0.57	31.4	102.4	2.4
	2	0.13	29.5	101.9	
	3	0.30	37.4	132.6	
					

## Data Availability

The X-ray data and the refined structure were submitted to the wwPDB (Protein Data Bank) with a deposition ID: D_1292118813 with an assigned accession code (s): PDB ID 7Q1L. SAXS data were submitted to and validated by SASBDB (https://www.sasbdb.org/) [50], with assigned accession codes: SASDMP7, SASDMN7, SASDMM7, SASDMS7, SASDMR7, SASDMM7 for apo- and holo-TF at pH 8.0, 7.0, and 5.5, respectively.

## Supplementary Materials

The following are available online at https://www.mdpi.com/article/10.3390/ijms222413392/s1.
