# Supplementary material for: X-ray Characterization of Conformational Changes of Human Apo- and Holo-Transferrin
Source: Int J Mol Sci. 2021 Dec 13;22(24):13392. doi: 10.3390/ijms222413392 (PMC8705962; doi:10.3390/ijms222413392)
Supplement: Supplementary file 1 [file ijms-22-13392-s001.zip › ijms-1467850-supplementary.pdf]

Supplementary Material

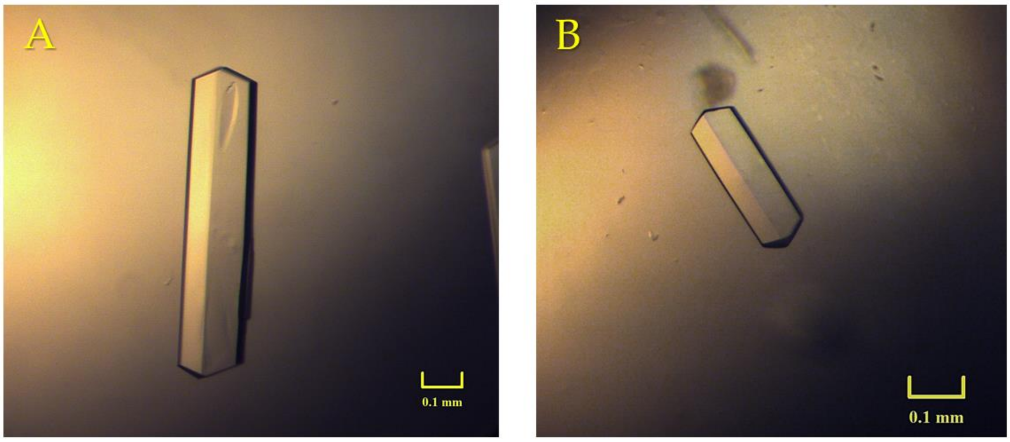

Figure S1 . Transferrin crystals obtained by vapor-diffusion. Both apo-Tf and holo-Tf were crystallized under the same conditions, at 20 mg mL^{-1} stock solution using Index screen #88 as precipitating agent (Hampton Research Corporation) at 18 °C. However, the crystal in B, grown from a holo-Tf solution, probably corresponds to apo-Tf as it appears to have lost its iron and looks very similar to the apo-Tf (A) crystal.

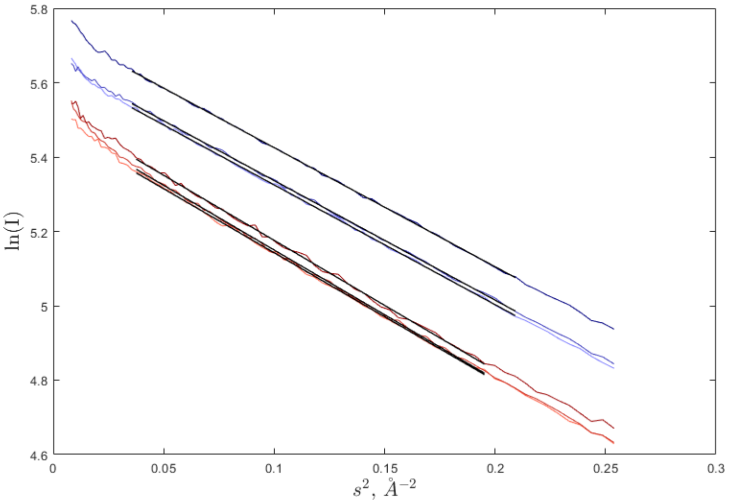

Figure S2 Guiner plot range and the linear fitting (black color).

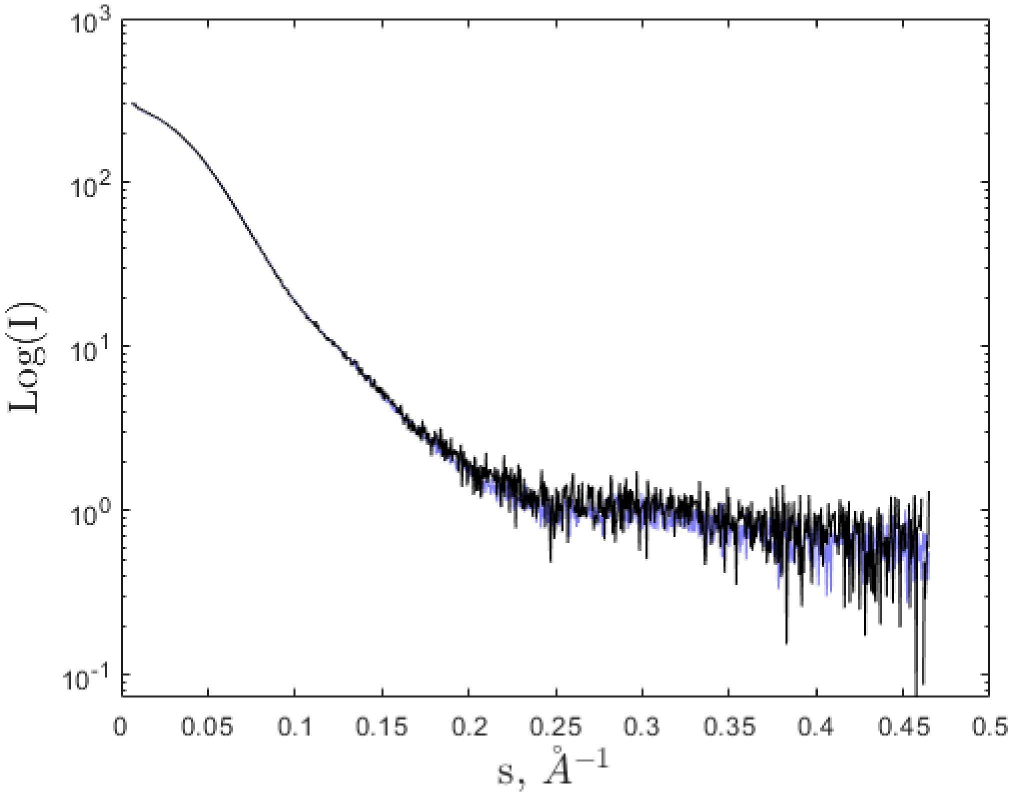

Figure S3. Experimental Log10 SAXS intensity versus scattering vector s, for apo-Tf pH 8.0 at concentration 5 mg mL (same color gradient used in Figure 2A) and 2.5 mg ml (black color)

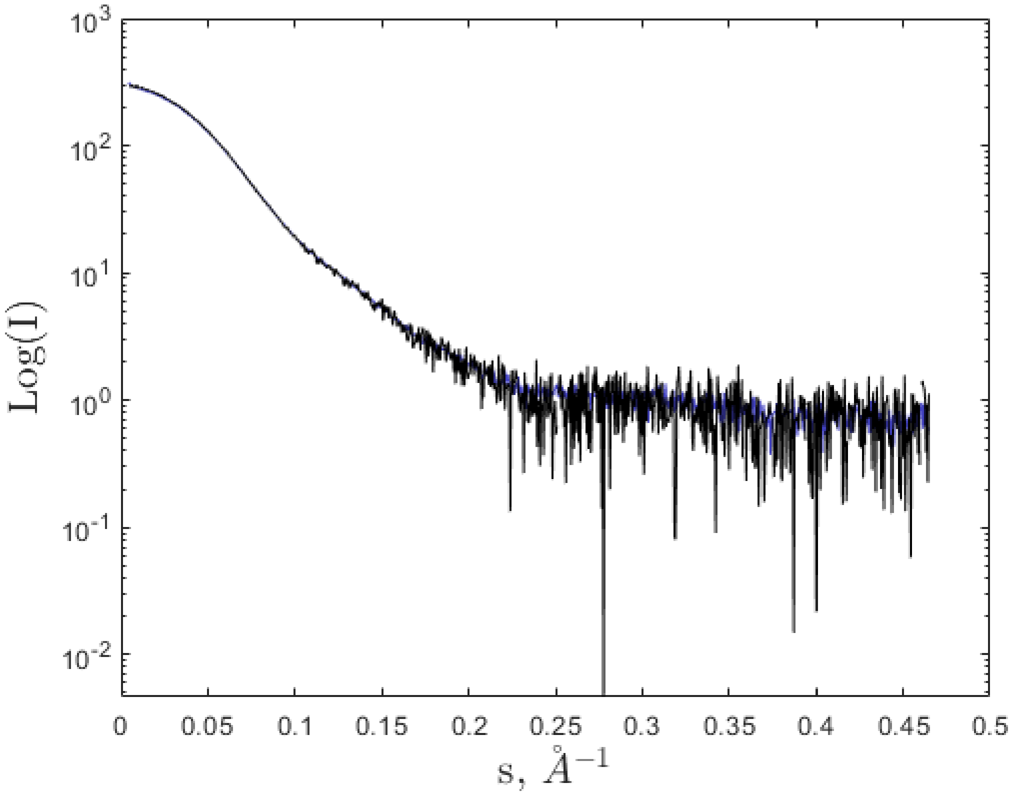

Figure S4. Experimental Log10 SAXS intensity versus scattering vector s, for apo-Tf pH 7.0 at concentration 5 mg mL (same color gradient used in Figure 2A) and 2.5 mg ml (black color)

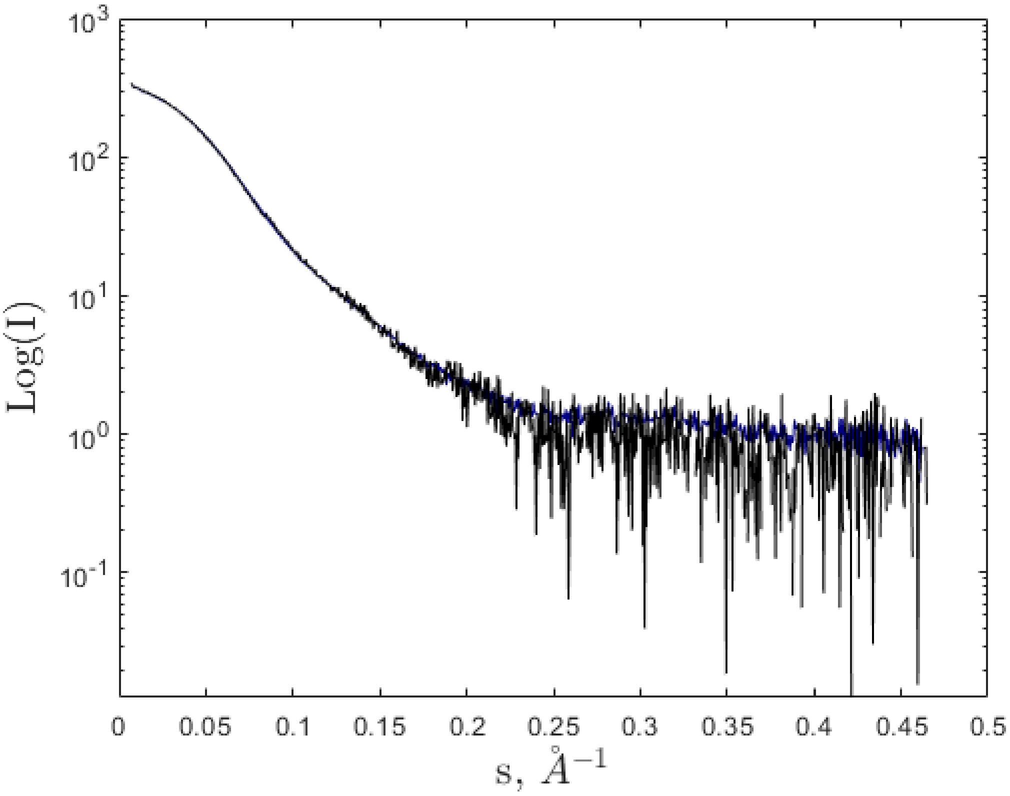

Figure S5. Experimental Log10 SAXS intensity versus scattering vector s, for apo-Tf pH 5.5 at concentration 5 mg mL (same color gradient used in Figure 2A) and 2.5 mg ml (black color)

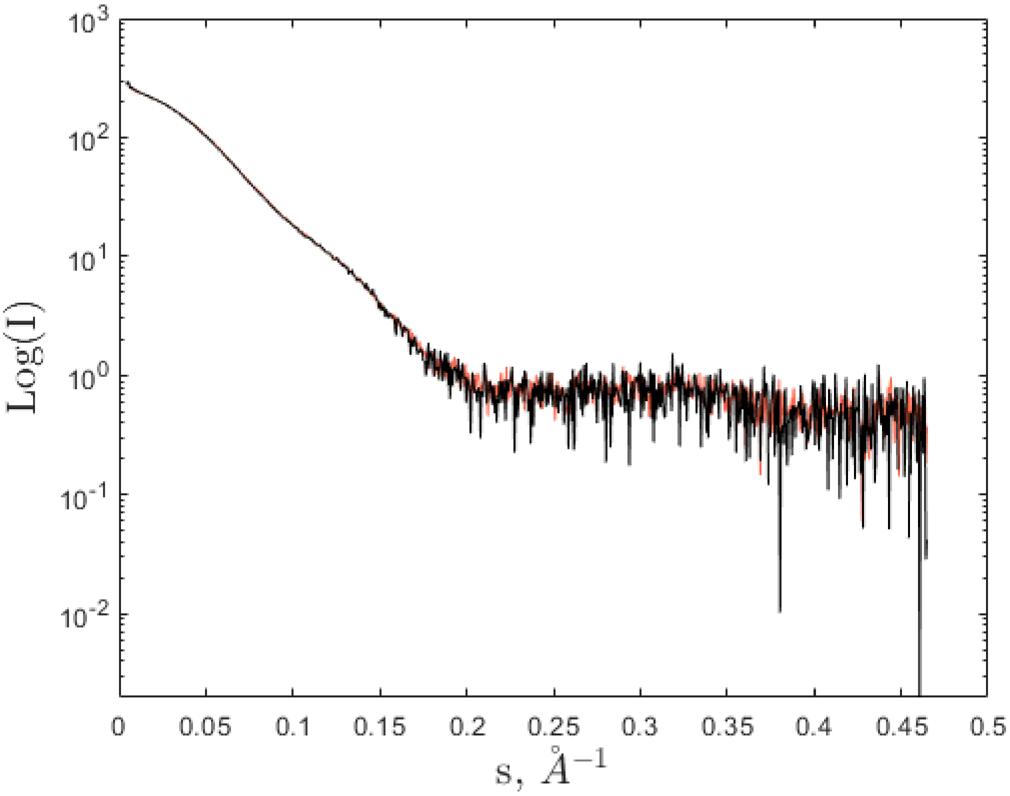

Figure S6. Experimental Log10 SAXS intensity versus scattering vector s, for holo-Tf pH 8.0 at concentration 5 mg mL (same color gradient used in Figure 2A) and 2.5 mg ml (black color)

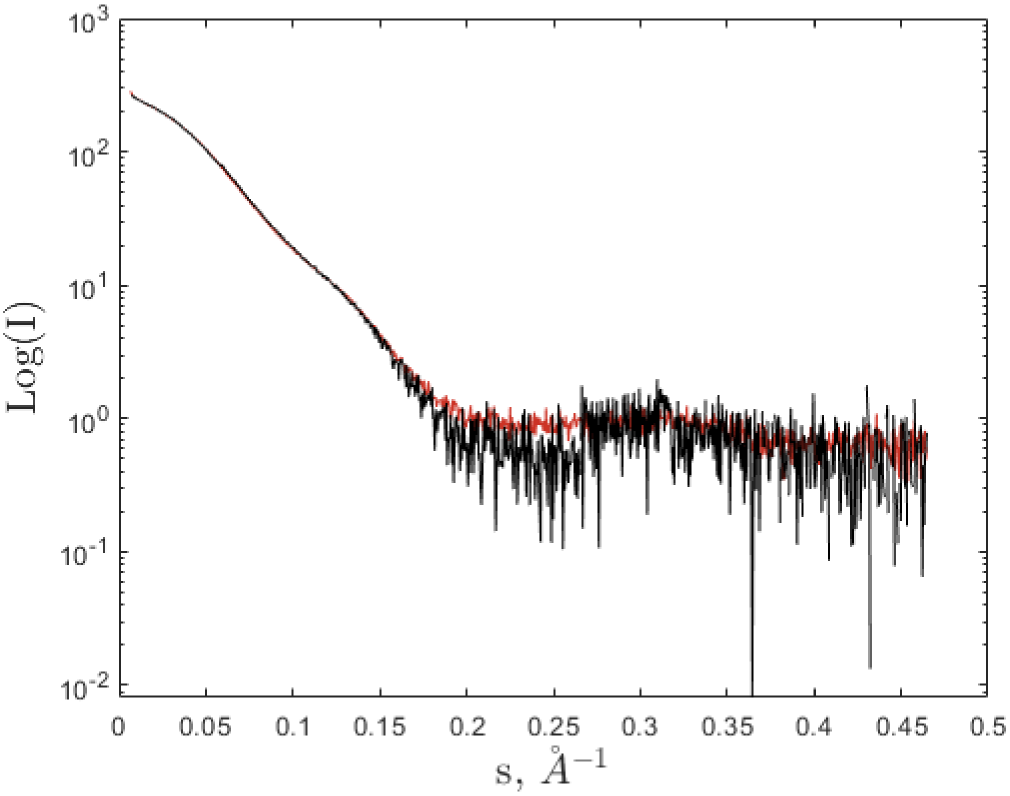

Figure S7. Experimental Log10 SAXS intensity versus scattering vector s, for holo-Tf pH 7.0 at concentration 5 mg mL (same color gradient used in Figure 2A) and 2.5 mg ml (black color)

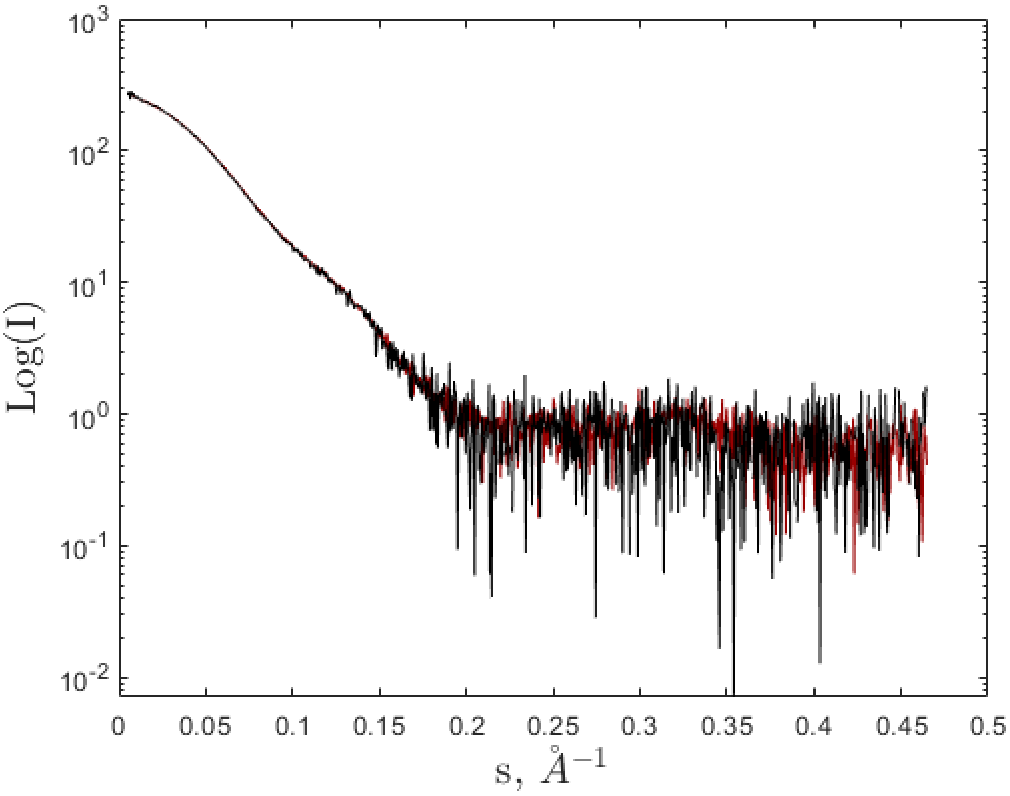

Figure S8. Experimental Log10 SAXS intensity versus scattering vector s, for holo-Tf pH 5.5 at concentration 5 mg mL (same color gradient used in Figure 2A) and 2.5 mg ml (black color)
